# Effects of Thiazolidinedione Therapy on Inflammatory Markers of Type 2 Diabetes: A Meta-Analysis of Randomized Controlled Trials

**DOI:** 10.1371/journal.pone.0123703

**Published:** 2015-04-21

**Authors:** Rui Chen, Jinchuan Yan, Peijing Liu, Zhongqun Wang

**Affiliations:** Department of Cardiology, Affiliated Hospital of Jiangsu University, Zhenjiang, Jiangsu Province, China; University of Texas Health Science Center at San Antonio, UNITED STATES

## Abstract

**Background:**

Inflammation is a common feature in patients with type 2 diabetes mellitus (T_2_DM). This meta-analysis aimed to assess the influence of thiazolidinedione (TZD) therapy on the circulating levels of inflammatory markers in patients with T_2_DM.

**Methods and Results:**

We searched the databases Medline, Embase, ScienceDirect, Web of Science, SpringerLink, and the Cochrane Library for randomized controlled trials (RCTs) that examined the effects of thiazolidinedione vs. a placebo on patients with T_2_DM. The main outcomes were absolute changes in levels of circulating inflammatory markers. Twenty-seven RCTs were included and data were analyzed using a fixed-effect model or a random-effect model based on heterogeneity. Pooled results indicated that circulating levels of high-sensitivity C reactive protein (hsCRP; SMD = –0.65, 95% CI = –0.98 to –0.32, *p* < 0.01), monocyte chemoattractant protein-1 (MCP-1; WMD = –54.19, 95% CI = –73.86 to –34.52, *p* < 0.01), von Willebrand factor% (vWF%; WMD = –8.18, 95% CI = –13.54 to –2.81, *p* 0.01), fibrinogen (SMD = –0.26, 95% CI = –0.41 to –0.11, *p* < 0.01) and E-selectin(WMD = –3.57, 95% CI = –5.59 to -1.54, *p* <0.01) were significantly decreased after TZD therapy. However, interleukin-6 (IL-6), matrix metalloproteinase-9 (MMP-9), soluble CD40 ligand, plasminogen activator inhibitor 1 (PAI-1) and intercellular adhesion molecule (ICAM-1) were not significantly affected. Subgroup analyses of PAI-1, vWF% and fibrinogen in terms of trial drugs showed significant reductions for rosiglitazone (all *p* valuses< 0.05), but not pioglitazone treatment. Conversely, the E-selectin (*p* < 0.01) lowering effect only existed in the pioglitazone group. Further, rosiglitazone and pioglitazone treatment reduced serum hsCRP and MCP-1 but had no marked effects on MMP-9, IL-6 and ICAM-1.

**Conclusions:**

Limited evidence suggested that TZD therapy had anti-inflammatory property that might contribute to its beneficial effect on inflammatory state in patients with type 2 diabetes.

## Introduction

Inflammatory state is a known contributor to the development of insulin resistance (IR) and vascular damage. The presence of activated circulating pro-inflammatory markers, such as high sensitivity C-reactive protein (hsCRP) and interleukin-6 (IL-6), systemic release of pro-thrombotic markers, such as plasminogen activator inhibitor 1 (PAI-1), and increased markers of endothelial dysfunction, such as E-selectin, are involved in the pathogenesis of vascular dysfunction [1–3] and IR [4,5]. Increased plasma concentrations of these inflammatory cytokines may indicate a significant increase in the risk of vascular damage and IR.

Peroxisome proliferator-activated receptor (PPAR) γ agonists, also called thiazolidinediones (TZDs), along with the derivatives pioglitazone and rosiglitazone, are among the main classes of oral anti-diabetic drugs. Currently, they are extensively used worldwide [6]. TZDs have shown potential retrogression for type 2 diabetes and prolonged glycemic control by increasing insulin sensitivity in the liver, muscles, and fat [7]. Studies have also focused on the improvement of vascular dysfunction [8,9]. Results of animal and large prospective trials have indicated that rosiglitazone and pioglitazone exhibit anti-inflammatory properties [10,11,12,13]. Considering that inflammatory processes are dysregulated in the pathogenesis of IR and vascular damage, we proposed that TZD therapy could improve IR and vascular damage by suppressing plasma inflammatory cytokines. However, the effects of TZD treatment on these molecules remain inconclusive. In the current study, a meta-analysis was performed using published data from randomized controlled trials (RCTs) to investigate the effects of TZD therapy on the serum levels of cytokines.

## Methods

### Search strategy

We conducted an online search using Medline, Embase, ScienceDirect, Web of Science, Springer Link, and the Cochrane Library from January 2000 to January 2015 without language restrictions. The terms used for this search were listed as follows: “thiazolidinediones;” “TZDs;” “peroxisome proliferator-activated receptor γ agonist;” “PPAR γ agonist;” “pioglitazone;” and “rosiglitazone.” These keywords were paired with the terms “inflammation,” “cardiovascular risk marker,” and “thrombotic marker.” The search was limited to clinical trials. The lists of original and review articles were then analyzed using a manual approach.

### Study selection

Studies were eligible for the present meta-analysis if they satisfy the following criteria: (1) human intervention studies with a prospective, randomized, and placebo-controlled trial (regardless of sample size); (2) analysis on adult patients with established type 2 diabetes and who were subjected to oral TZD (pioglitazone or rosiglitazone) therapy or placebo (we adopted the criteria established by the World Health Organization and the American Diabetes Association for the diagnosis of type 2 diabetes: fasting glucose >126 mg/dl (7.0 mmol/L) or 2 h blood glucose >200 mg/dl (11.1 mmol/L); (3) at least one of the following circulating cardiovascular risk markers was included and allowed calculation of the net change: hsCRP, matrix metalloproteinase-9 (MMP-9), monocyte chemoattractant protein (MCP)-1, IL-6, soluble CD40 ligand (sCD40L), von Willebrand factor% (vWF%), PAI-1, fibrinogen, E-selectin, and intercellular adhesion molecule (ICAM)-1; and (4) full-length articles.

### Data extraction and quality assessment

Data were extracted by two authors, and results were compiled. Disagreement was resolved by consensus or an opinion of a third author if necessary. The following data were extracted: baseline characteristics (lead author, publication year, study design, sample size, and mean age) and treatment regimen (dose of pioglitazone or rosiglitazone, composition of placebo and intervention duration). If the study provided interquartile ranges (IQRs) and medians instead of means ± standard deviations (SDs), we assigned the means ± SDs as previously described [14].

The quality of the studies was assessed on the basis of randomization procedures, random number generation, double-blinding procedures, information on withdrawals, and allocation concealment. Studies were scored 1 point for each of the addressed areas ranging from 0 to 5 points. High-quality RCTs scored ≥3 points whereas low-quality RCTs scored <3 points based on a modified Jadad score.

### Statistical analysis

All of the endpoints were estimated on the basis of the mean absolute changes from the baseline. The significance of the net changes was calculated using weight mean difference (WMD) or standardized mean difference (SMD) and 95% confidence interval (CI) with a fixed-effect model or a random-effect model. The heterogeneity of intervention effects among the studies was evaluated by Cochrane’s test. Significant heterogeneity was considered if *P* <0.1. *I* ^2^ statistic was also examined, where *I* ^2^ of 25%, 50%, and 75% indicated low, moderate, and high degrees of heterogeneity, respectively. Publication bias was assessed by using funnel plots and Egger’s test. The software RevMan (version 5.1; Cochrane Collaboration) and Stata (version 10.0; Stata Corporation) supported the analysis. Subgroup analysis was performed on the basis of the types of trial drug.

## Results

### Search results

**Fig 1:**
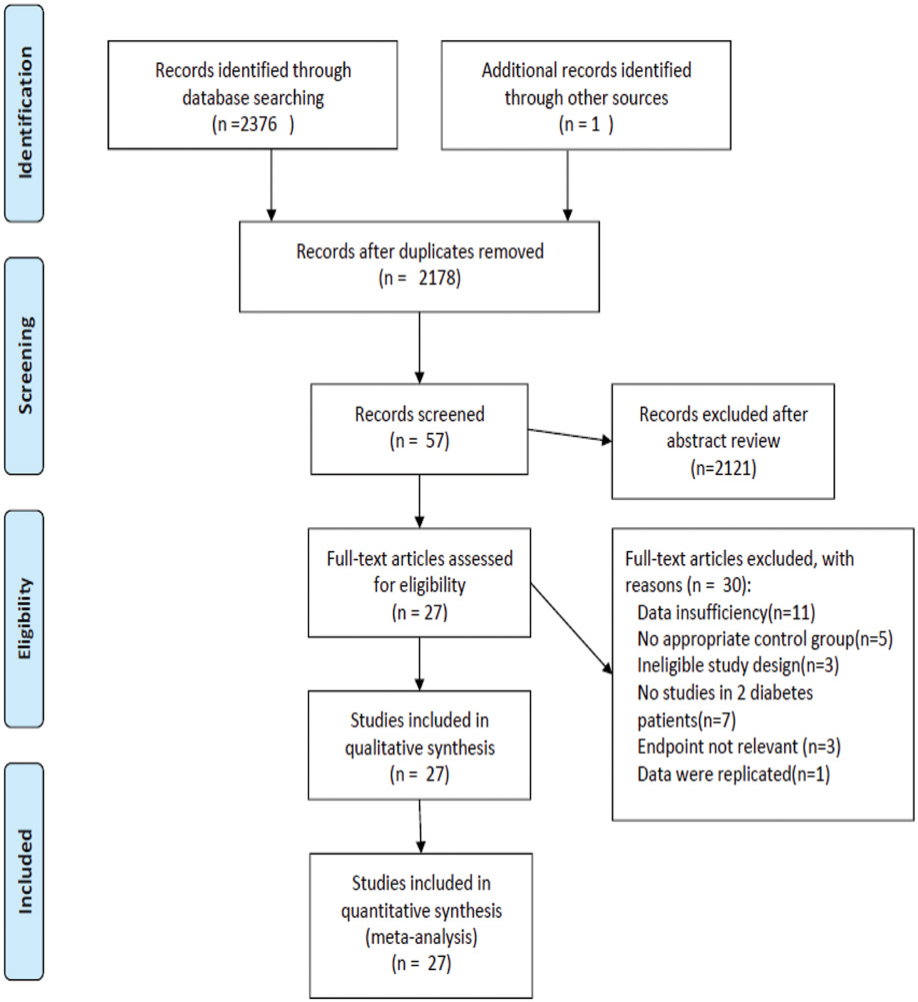
Process of study selection.

A total of 2,377 studies were initially identified; among these studies, 2,320 were excluded after titles and abstracts were screened. The full texts of the 57 remaining studies were analyzed. Among these 57 studies, 30 were excluded because of the following reasons: first, 11 studies provided insufficient data on related outputs; second, 5 studies did not include an appropriate control group; third, 3 studies used an ineligible study design; fourth, participants in 7 studies were not patients with type 2 diabetes. Finally, the endpoints were not relevant in 3 studies and 1 study reported replicated data (Fig 1).

### Study characteristics

A total of 27 RCTs, including 5 open-labeled randomized trials (19, 25, 28, 38 and 40), were eligible for the present meta-analysis (Table 1). 11 of the observational studies (15, 16, 19, 21–24, 29, 32, 36 and 38) were used rosiglitazone as the primary source of TZDs; in the other studies (17, 18, 20, 25–28, 30, 31, 33–35, 37, 39–41), pioglitazone was used. Pioglitazone and rosiglitazone doses varied from 15 mg/d to 45 mg/d and from 4 mg/d to 8 mg/d, respectively. Among the 27 studies, 7 investigated the effects of TZDs on type 2 diabetic patients with coronary artery disease (15, 16, 20, 27, 28, 29, and 33). 12 studies examined type 2 diabetic subjects (19, 21, 22, 24, 30–32 and 36–40) only, and the remaining studies were conducted on individuals with a combination of type 2 diabetes and one of the following conditions: dislipidemia (17); obesity (18 and 35); asymptomatic carotid stenosis (23 and 34); chronic kidney disease (24); atherosclerosis (26) and kidney transplant(41).

**Table 1:** Overview and characteristics of included studies.

study	NO. of patients		mean age (y)		population	dosage	duration	design	Medication studied		J score
	exposed group	control group	exposed group	control group		mg/d					
**Bertrand 2010**	**98**	**95**	**64.2±7.3**	**65.1±6.9**	**T**_**2**_ **DM with CAD**	**8**	**12m**	**Ra, DB PC**	**R**	**not TZDs use**	**5**
**Finn 2009**	**32**	**33**	**65.7±9.6**	**59.7±9.3**	**T**_**2**_ **DM with CAD**	**4**	**8m**	**Ra, DB,PC**	**R**	**metformin**	**4**
**Schondorf 2011**	**25**	**21**	**59.4±8.0**	**57.5±10.1**	**T**_**2**_ **DM with dislipidimia**	**15**	**24w**	**Ra,DB,PC**	**P(+metformin)**	**Glimepiride(+metformin)**	**3**
**Harteman 2009**	**14**	**13**	**62±10**	**58±10**	**T**_**2**_ **DM with obesity**	**30**	**24w**	**Ra, PC**	**P(+sulfonylurea+metformin)**	**insule(+sulfonylurea+metformin)**	**5**
**Kiyici 2009**	**19**	**16**	**50.7±6.4**	**52.4±8.3**	**T**_**2**_ **DM**	**4**	**52w**	**Ra,PCopen-label**	**R**	**metformin**	**3**
**Forst 2008**	**44**	**48**	**64.5±7.3**	**65.7±7.5**	**T**_**2**_ **DM with CAD**	**45**	**4w**	**Ra,DB,PC**	**P**	**not TZDs use**	**3**
**Davidson 2007**	**117**	**116**	**52±11.9**	**53±10.4**	**T**_**2**_ **DM**	**8**	**24w**	**Ra,DB,PC**	**R(+Glimepiride)**	**not TZDs use(+Glimepiride)**	**4**
**Fidan 2011**	**20**	**20**	**54.1±9.0**	**52.6±7.2**	**T**_**2**_ **DM**	**4/8**	**12w**	**Ra,PC**	**R**	**metformin**	**3**
**Marfella 2006**	**23**	**23**	**69**±**2**	**68±3**	**T**_**2**_ **DM with asymptomatic carotid stenosis**	**8**	**4m**	**Ra,DB,PC**	**R(+sulfonylurea +metformin+insuline)**	**not TZDs use (**_**+**_ **sulfonylurea+ metformin+insuline)**	**3**
**Chan 2011**	**35**	**35**	**62±10**	**62±10**	**T**_**2**_ **DM with chronic kidney disease**	**4**	**8w**	**Ra,DB,PC**	**R**	**not TZDs use**	**4**
**Pfuttzner 2005**	**89**	**84**	**62.2±8.4**	**63.0±7.4**	**T**_**2**_ **DM**	**45**	**26±2w**	**Ra,PC open-label**	**P**	**glimepiride**	**3**
**Mizoguchi 2011**	**31**	**21**	**68.2±7.3**	**68.0±9.1**	**T**_**2**_ **DM with atherosclerosis**	**30**	**4m**	**Ra,PC**	**P**	**glimepiride**	**3**
**Hong 2010**	**47**	**47**	**63.5±7.4**	**62.4±8.3**	**T**_**2**_ **DM with CAD**	**30**	**8m**	**Ra,SB,PC**	**P**	**not TZDs use**	**3**
**Oqasawara 2009**	**22**	**24**	**68.6±7.9**	**66.8±8.1**	**T**_**2**_ **DM with CAD**	**15**	**6m**	**Ra,PC open-label**	**P**	**not TZDs use**	**3**
**Yu 2007**	**25**	**21**	**63.6±11.2**	**63.5±9.8**	**T**_**2**_ **DM with CAD**	**4**	**12w**	**Ra,PC**	**R**	**not TZDs use**	**3**
**Hanefeld 2011**	**40**	**42**	**61.5±7.1**	**64.2±7.3**	**T**_**2**_ **DM**	**30**	**6m**	**Ra,DB,PC**	**P(+insulin)**	**Metformin(+insulin)**	**3**
**Yener 2009**	**20**	**20**	**51.7±8.1**	**53.2±8.0**	**T**_**2**_ **DM**	**4**	**3m**	**Ra,PC**	**P**	**metformin**	**3**
**Kelly 2007**	**20**	**16**	**57.9±8.2**	**63.1±7.8**	**T**_**2**_ **DM**	**8**	**6m**	**Ra,DB,PC**	**R(+metformin)**	**Glyburide(+metformin)**	**3**
**Nissen 2008**	**270**	**273**	**60.0±9.4**	**59.7±9.1**	**T**_**2**_ **DM with CAD**	**45**	**18m**	**Ra,DB,PC**	**P**	**glimepiride**	**5**
**Langenfeld 2005**	**89**	**84**	**62±8**	**63±7**	**T**_**2**_ **DM with asymptomatic carotid stenosis**	**45**	**24±4w**	**Ra,PC**	**P**	**glimepiride**	**3**
**Tripathy 2013**	**11**	**9**	**56±2.8**	**57±2.2**	**T**_**2**_ **DM with obesity**	**15**	**6m**	**Ra, DB PC**	**P(+sulfonylurea +metformin)**	**not TZDs use (**_**+**_ **sulfonylurea+ metformin)**	**5**
**Busui 2009**	**14**	**13**	**49.5±10**	**49.5±10**	**T**_**2**_ **DM**	**8**	**6m**	**Ra,PC**	**R**	**glyburide**	**3**
**Derosa 2010**	**138**	**136**	**55±8**	**57±6**	**T**_**2**_ **DM**	**45**	**3m**	**Ra, DB PC**	**P**	**acarbose**	**5**
**Stocker 2007**	**37**	**38**	**64±11**	**65±10**	**T**_**2**_ **DM**	**4**	**24w**	**Ra,PCopen-label**	**R**	**metformin**	**3**
**Martens 2006**	**8**	**8**	**57±2**	**55±3**	**T**_**2**_ **DM**	**30**	**4w**	**Ra, DB PC**	**P**	**not TZDs use**	**5**
**Agarwal 2006**	**21**	**19**	**67±8.5**	**64±8.4**	**T**_**2**_ **DM**	**33±10**	**16w**	**Ra,PCopen-label**	**P**	**glimepiride**	**3**
**Kharazmkia 2014**	**31**	**31**	**50.2±12.6**	**54.8±8.7**	**T**_**2**_ **D with kidney transplant**	**30**	**4m**	**Ra, DB PC**	**P**	**not TZDs use**	**5**

TZDs = thiazolidinediones, P = pioglitazone, R = rosiglitazone, Ra = random, DB = double bind, SB = single blind, PC = placebo-controlled, T_2_DM = type 2 diabetes,CAD = coronary artery disease.

### Data quality

The quality scores (Table 1) of these RCTs varied from 3 to 5 (maximum score). A total of 10 studies were classified as high quality with a Jadad score of 4 or 5 (15, 16, 18, 21, 24, 33, 35, 37, 39 and 41) and 17 studies yielded a Jadad score of 3 (17, 19, 20, 22, 23, 25–32, 34, 36, 38 and 40).

### Effects of TZD therapy on the plasma concentrations of pro-inflammatory markers

**Fig 2:**
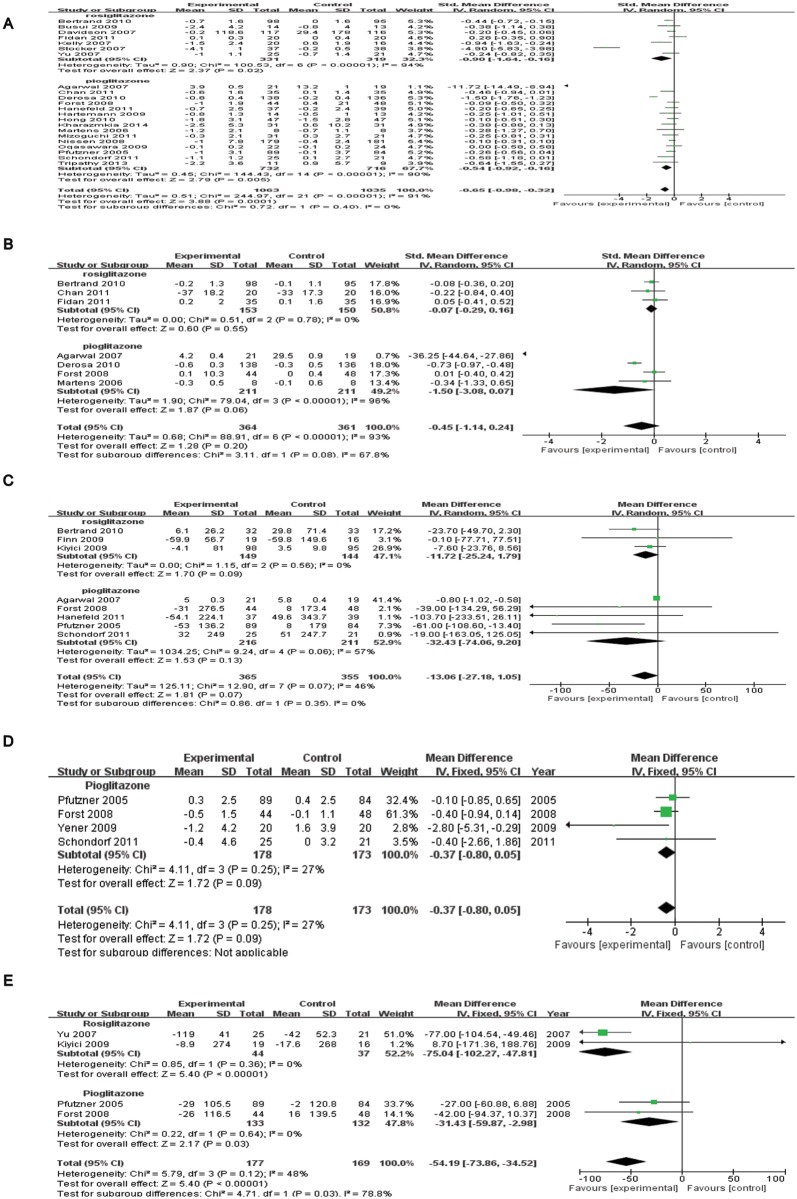
Forest plots from meta-analysis of RCTs regarding the role of thiazolidinedinediones therapy in plasma concentrations of pro-inflammatory markers hsCRP(A), IL-6(B), MMP-9(C), sCD40L(D) and MCP-1(E).

22 studies with 2098 patients reported the effects of TZD therapy on hsCRP levels (SMD = -0.65, 95% CI = -0.98 to -0.32, *p* <0.01; heterogeneity test: chi-square = 244.97, *I* ^2^ = 91%, *p* <0.01; Fig 2A). The hsCRP-lowering effect was consistent in both subgroups: rosiglitazone [subtotal SMD = -0.90, 95% CI = -1.64 to -0.16, *p* = 0.02, *I* ^2^ = 94%, 7 trials, *n* = 650] and pioglitazone [Subtotal SMD = -0.54, 95% CI = -0.92 to -0.16, *p* <0.05, *I* ^2^ = 90%, 15 trials, *n* = 1448]. Five studies were excluded for sensitivity analysis because of the following reasons: hsCRP data were imputed from median [15], IQRs [16], and three open-labeled studies [17, 18 and 19]. It also suggested a significant lowing effect (SMD = -0.36, 95% CI = -0.62 to -0.09, *p <*0.01).

In a pooled analysis of seven studies with 725 patients, the circulating levels of IL-6 were not significantly reduced (SMD = –0.45, 95% CI = –1.14 to 0.24, *p* = 0.20; heterogeneity test: chi-square = 88.91, *I* ^2^ = 93%, *p*<0.01; Fig 2B). The IL-6 lowering effect was not observed in both subgroups: rosiglitazone [Subtotal SMD = –0.07, 95% CI = –0.29 to 0.16, *I* ^2^ = 0%, *p* = 0.55, 3 trials, *n* = 303] and pioglitazone [Subtotal SMD = -1.5, 95% CI = –3.08 to 0.07, *p* = 0.06, 4 trials, *n* = 422]. Three studies were excluded for sensitivity analysis because IL-6 data were imputed from median [15] and IQRs [19, 20]. The result of the sensitivity analysis indicated that these three studies had no effect (SMD = -0.26, 95% CI = –0.75 to 0.23, *p* = 0.29).

Eight studies with 720 patients were pooled in terms of MMP-9 (WMD = –13.06, 95% CI = –27.18 to 1.05, *p* = 0.07; heterogeneity test: chi-square = 12.90, *I* ^2^ = 46%, *p* = 0.07; Fig 2C). There was no significant reduction in both subgroups: rosiglitazone [Subtotal WMD = –11.72, 95% CI = –25.24 to 1.79, *I* ^2^ = 0%, *p* = 0.09, 3 trials, *n* = 293] and pioglitazone [Subtotal WMD = –32.43, 95% CI = –74.06 to 9.20, *I* ^2^ = 57%, *p* = 0.13, 5 trials, *n* = 427]. Three studies were excluded for sensitivity analysis because of the following reasons: MMP-9 data were imputed from median [15] and two open-labeled studies [17, 19]. They made no difference to the result of TZDs treatment (WMD = –9.61, 95% CI = –25.01 to 5.80, *p* = 0.22).

No significant reduction was found in sCD40 L concentration (WMD = –0.37, 95% CI = –0.80 to 0.05, *p* = 0.09; heterogeneity test: chi-square = 4.11, *I* ^2^ = 27%, *p* = 0.25; Fig 2D) based on the pooled results of four studies with 351 patients who were only in the pioglitazone group. The result of the sensitivity analysis, excluding one study in which sCD40L data were imputed from an open-labeled study [17], was not significant either (WMD = –0.50, 95% CI = –1.02 to 0.01, *p* = 0.06).

All of the four studies with 346 patients were pooled in terms of MCP-1 (WMD = -54.19, 95% CI = -73.86 to -34.52, *p*<0.01; heterogeneity test: chi-square = 5.79, *I* ^2^ = 48%, *p* = 0.12; Fig 2E). The lowering effect was consistent in both subgroups: rosiglitazone [Subtotal WMD = -75.04, 95% CI = -102.27 to -47.81, *I* ^2^ = 0%, *p*<0.01, 2 trials, *n* = 81] and pioglitazone [Subtotal WMD = -31.43, 95% CI = -59.87 to -2.98, *I* ^2^ = 0%, *p* = 0.03, 2 trials, *n* = 265]. The sensitivity analysis, excluding one study in which MCP-1 data were imputed from an open-labeled study [17], did not affect the result (WMD = -68.01, 95% CI = -92.17 to -43.85, *p*<0.01).

### Effects of TZD therapy on the plasma concentrations of pro-thrombotic markers

**Fig 3:**
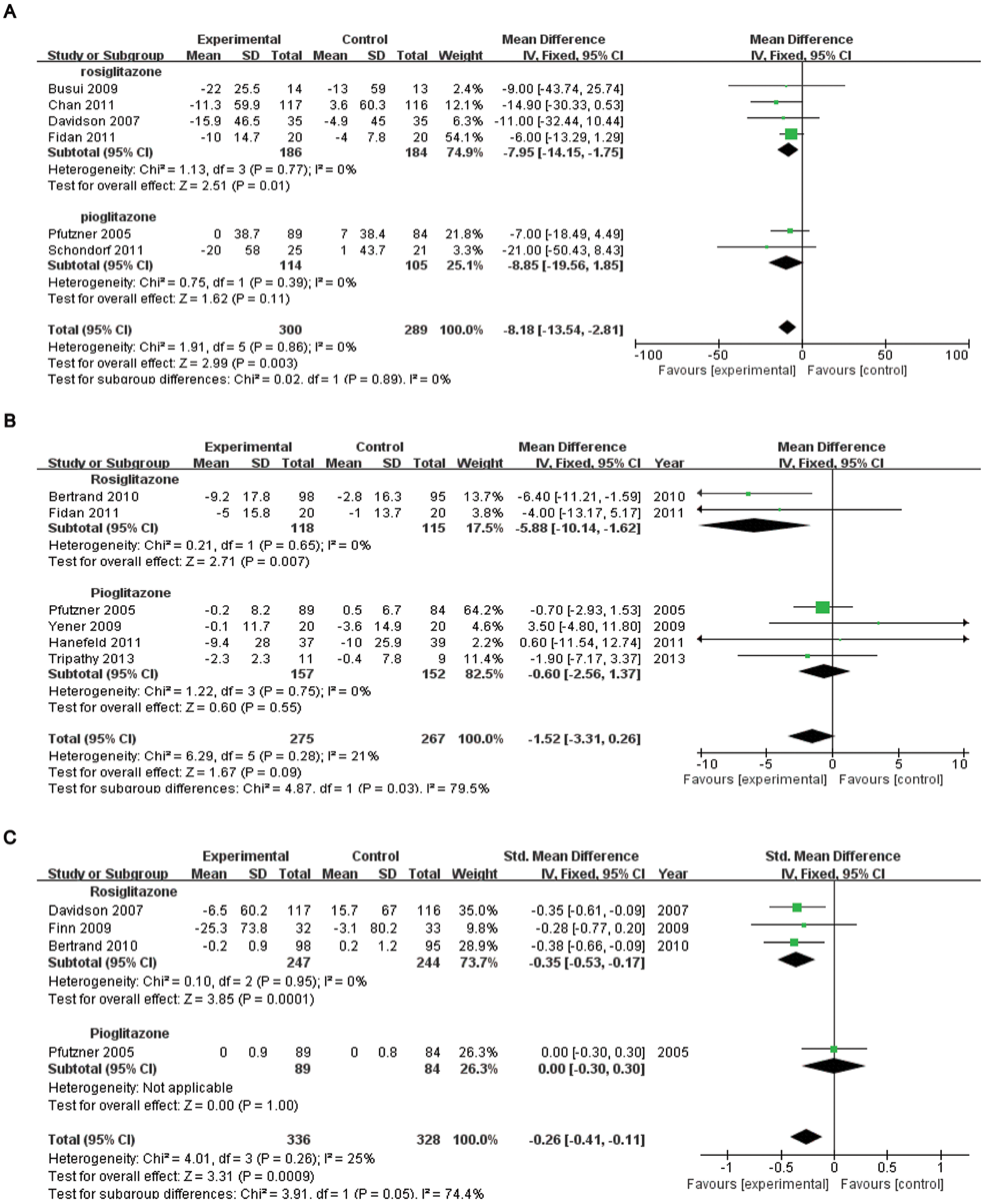
Forest plots from meta-analysis of RCTs regarding the role of thiazolidinedinediones therapy in plasma concentrations of pro-thrombotic markers, vWF%(A), PAI-1(B)and fibrinogen(C).

Six studies with 589 patients reported the effects of TZD therapy on vWF% levels (WMD = -8.18, 95% CI = -13.54 to -2.81, *p*<0.01; heterogeneity test: chi-square = 1.91, *I* ^2^ = 0.0%, *p* = 0.86; Fig 3A). However, differences were observed between the subgroups rosiglitazone [Subtotal WMD = -7.95, 95% CI = -14.15 to -1.75, *I* ^2^ = 0%, *p* = 0.01, 4 trials, *n* = 370] and pioglitazone [Subtotal WMD = –8.85, 95% CI = –19.56 to 1.85, *I* ^2^ = 0%, *p* = 0.11, 2 trials, *n* = 219]. The sensitivity analysis, excluding two studies in which vWF% data were imputed from an open-labeled study [17] and IQRs [21], did not affect the result (WMD = -8.29, 95% CI = -14.62 to -1.96, *p* = 0.01).

No significant reduction was found in the PAI-1 concentrations (WMD = –1.52, 95% CI = –3.31 to 0.26, *p* = 0.09; heterogeneity test: chi-square = 6.29, *I* ^2^ = 21%, *p* = 0.28; Fig 3B) based on the pooled results from six studies with 542 patients. However, different effects were observed between the subgroups rosiglitazone [Subtotal WMD = -5.88, 95% CI = -10.14 to -1.62, *I* ^2^ = 0%, *p*<0.01, 2 trials, *n* = 233] and pioglitazone [Subtotal WMD = –0.60, 95% CI = –2.56 to 1.37, *I* ^2^ = 0%, *p* = 0.55, 4 trials, *n* = 309]. The sensitivity analysis, excluding two studies in which PAI-1 data were imputed from an open-labeled study [17] and median [15], suggested that the effect of TZDs therapy was not significant either (WMD = –0.88, 95% CI = –4.69 to 2.92, *p* = 0.65).

Four studies with 664 patients were pooled in terms of fibrinogen (SMD = -0.26, 95% CI = -0.41 to -0.11, *p*<0.01; heterogeneity test: chi-square = 4.01, *I* ^2^ = 25%, *p* = 0.26; Fig 3C). However, when it comes to the subgroup analysis, different results were observed: rosiglitazone [Subtotal SMD = -0.35, 95% CI = -0.53 to -0.17, *I* ^2^ = 0%, *p*<0.01, 2 trials, *n* = 491] and pioglitazone [Subtotal SMD = 0, 95% CI = –0.30 to 0.30, 1 trial, *n* = 173].

### Effects of TZD therapy on plasma concentrations of adhesion molecules

**Fig 4:**
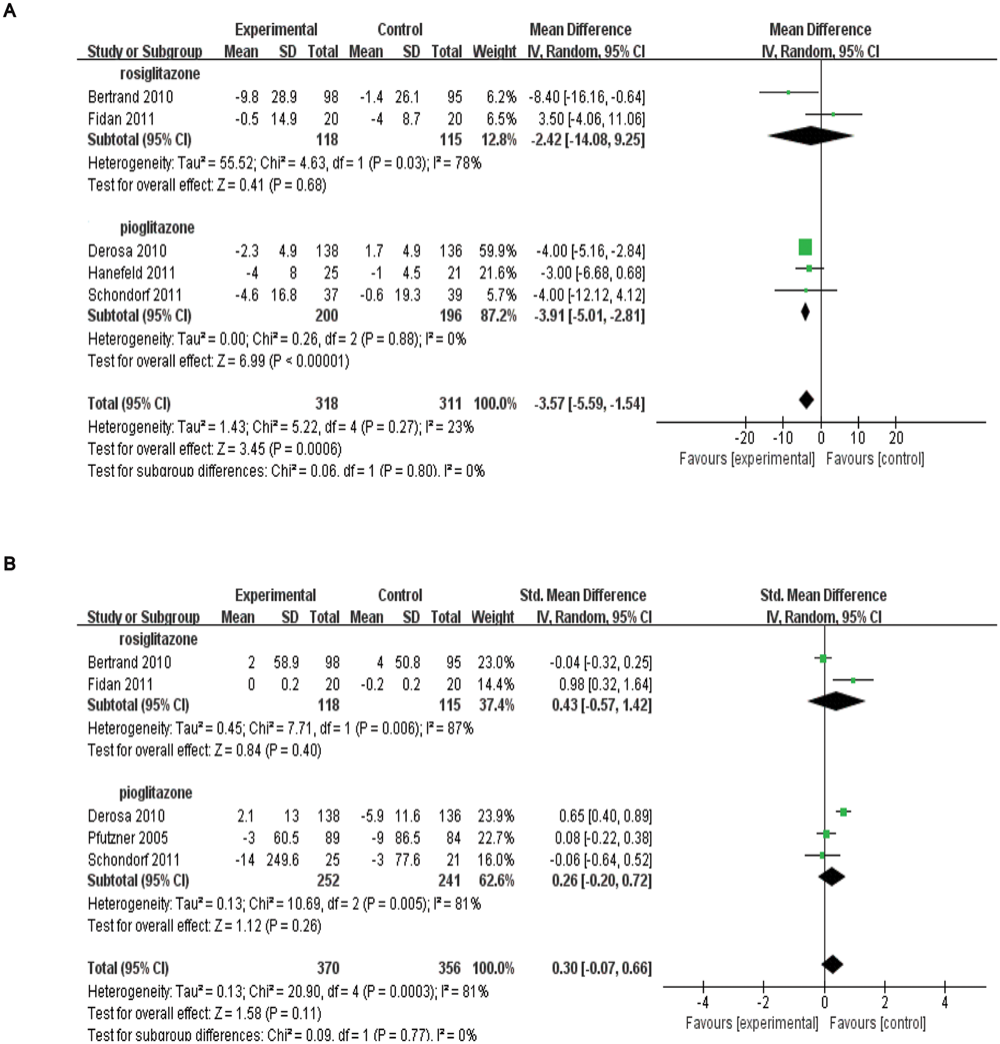
Forest plots from meta-analysis of RCTs regarding the role of thiazolidinedinediones therapy in plasma concentrations of adhesion molecules, E-selectin(A) and ICAM-1(B).

All of the five studies with 629 patients reported the effects of TZD therapy on E-selectin levels (WMD = –3.57, 95% CI = –5.59 to -1.54, *p<*0.01; heterogeneity test: chi-square = 5.22, *I* ^2^ = 23%, *p* = 0.27; Fig 4A). The effects were not consistent when the subgroup analysis based on different trial drugs were considered: rosiglitazone [Subtotal WMD = –2.42, 95% CI = –14.08 to 9.25, *I* ^2^ = 78%, *p* = 0.68, 2 trials, *n* = 233] and pioglitazone [Subtotal WMD = –3.91, 95% CI = –5.01 to -2.81, *I* ^2^ = 0%, *p*<0.01, 3 trials, *n* = 396].

No significant reduction was determined in the ICAM-1 concentration (SMD = 0.30, 95% CI = –0.07 to 0.66, *p* = 0.11; Fig 4B). The effects of the subgroup analysis were consistent: rosiglitazone [Subtotal SMD = 0.43, 95% CI = –0.57 to 1.42, *I* ^*2*^ = 87%, *p* = 0.40, 2 trials, *n* = 233] and pioglitazone [Subtotal SMD = 0.26, 95% CI = –0.20 to 0.72, *I* ^2^ = 81%, *p* = 0.26, 3 trials, *n* = 493]. The sensitivity analysis, excluding one study in which ICAM-1 data were imputed from an open-labeled study [17], revealed a not significant effect either (SMD = 0.40, 95% CI = –0.03 to 0.82, *p* = 0.07).

### Publication bias

The funnel plot appeared symmetric by visual inspection (S1 Fig). We also performed Egger’s test to check for a potential publication bias. No evidence of publication bias was found for the outcomes of hsCRP (*p* = 0.152). Publication bias investigation was not performed for other inflammatory markers owing to the limited numbers of the included studies.

## Discussion

The present meta-analysis included 27 trials which investigated the effects of TZD therapy on the plasma concentrations of cytokines in patients with type 2 diabetes. Significant reductions in the majority of these cytokines, such as hsCRP, MCP-1, vWF%, fibrinogen and E-selectin were observed in the TZD group compared with those of the placebo group among patients with type 2 diabetes. The levels of IL-6, MMP-9, sCD40L, PAI-1 and ICAM-1 showed no significant change. In the subgroup analyses based on different trial drugs, the lowering effects of PAI-1, vWF%, and fibrinogen were only observed in the rosiglitazone group. E-selectin level was significantly decreased only in the pioglitazone group. The serum hsCRP and MCP-1 revealed marked reductions in each group. There was no remarkable change in plasma IL-6, MMP-9 and ICAM-1 in both groups.

### Cytokines, TZDs, and diabetic vascular damage

Inflammatory processes and immune mechanisms have been associated with endothelial dysfunction, plaque progression, and acute coronary syndromes. A marked inflammatory activation and an increased level of thrombotic markers play an important role in the initiation and progression of atherosclerotic lesions [22]. Furthermore, this increased level of pro-inflammatory markers is the major cause of vascular dysfunction in diabetes [23]. High glucose levels increase the expression of pro-inflammatory cytokine and chemokine genes in monocytes. Therefore, inflammation may be a treatment target for patients with type 2 diabetes and vascular complications.

TZDs are insulin-receptor sensitive substances and function by activating PPAR γ. TZD therapy may affect cardiovascular risk factors, including pro-inflammatory markers, pro-thrombotic markers, and adhesion molecules in patients with or without type 2 diabetes [15–21, 24–43]. We systematically evaluated the effects of TZD therapy on the circulating levels of cardiovascular risk markers in patients with type 2 diabetes because the serum levels of these markers primarily function in cardiovascular disorders. In our meta-analysis, rosiglitazone elicited favorable effects on certain pro-inflammatory markers (hsCRP and MCP-1) and all of the pro-thrombotic markers (vWF%, PAI-1, and fibrinogen). Consistent with other studies, our study revealed that PPAR γ-activators inhibited inflammation that may contribute to the reduction of cardiovascular events in diabetic patients [44]. Regarded as one of the independent predictors of cardiovascular disease, hsCRP mediates pro-inflammatory and pro-atherogenic effects in endothelial cells, leading to vascular damage [45]. In the cardiovascular system, increased PAI-1, fibrinogen, MCP-1, and vWF% levels can result in slow fibrinolysis and fibrin buildup, supporting the initiation and propagation of thrombosis, instability of atheromatous plaques, and aggravation of platelet adhesion and aggregation [46]. Tousoulis et al. [47] reported that myocardial infarction is accompanied by an increased inflammatory process, as well as increased thrombotic and impaired fibrinolytic activities. These results suggested that the suppression of inflammatory markers in circulation may be involved in the improvement of vascular function of patients with type 2 diabetes treated with pioglitazone and rosiglitazone. Although Gada et al. [48] found that rosiglitazone adversely affects three novel biomarkers, namely, lymphotoxin β receptor, peptidoglycan recognition protein 1, and chemokine ligand 23, they failed to show that such complex effects could directly lead to vascular dysfunction. An increase in circulating MMP-9 contributes to acute coronary syndrome by destabilizing the protective fibrous caps of plaques [46]. However, in our meta-analysis, the reduction of MMP-9 level could not be observed in both subgroups. TZD therapy also did not show significant effects on the circulating levels of IL-6, and ICAM-1; these results were consistent with those of the subgroup analyses. This finding may be attributed to the following: first, TZDs may selectively lower the plasma concentrations of these cytokines rather than all of them and second, limited studies and small population size may fail to show favorable effects.

### Cytokines, TZDs, and IR

IR is an important pathophysiological characteristic of T_2_DM not only for hyperinsulinemia but also for its association with chronic inflammation. IR is partially transmitted by low-grade inflammation. High levels of cytokines, such as IL-6, TNF-α, CRP, and PAI-1, may result in increased IR via various molecular mechanisms [49, 50, 51]. As potent PPAR-γ activators, TZDs improve insulin sensitivity by regulating the expressions of several proteins in the insulin-signaling pathway. Our results failed to show any modification of IL-6 after rosiglitazone or pioglitazone treatment. This result was observed possibly because approximately 15% to 35% of the total circulating IL-6 is produced by adipocytes, which likely mediate insulin activity mainly by adiposity [51]. However, the study population of IL-6 in our results comprises non-obese individuals who exhibit a lower baseline. Rosiglitazone could reduce serum PAI-1 and hsCRP levels. Our results suggested that the modification of IR by TZDs may partially result from its anti-inflammatory effect.

The limitations of our meta-analysis are presented as follows. First, many of these trials used a small sample size and conducted for a short period; the confidence intervals for several cardiovascular risk markers were wide, resulting in inconsiderable uncertainties regarding the magnitude of the observed reduction. Second, health status, lifestyle, and basic oral anti-diabetic treatment were different among the subjects and may be an important source of heterogeneity. Third, we did not have access to the original data source related to individual participants. Moreover, a meta-analysis is deficient in authority compared with a large prospective trial designed to evaluate the outcome of interest.

Despite these limitations, our meta-analysis that pooled the limited number of RCTs indicated that rosiglitazone and pioglitazone therapy administered to patients with type 2 diabetes could reduce the circulating concentrations of several specific inflammatory markers. These results suggested that TZD treatment of patients with type 2 diabetes could elicit anti-inflammatory effect.

## Supporting Information

S1 PRISMA Checklist PRISMA Checklist. (DOC)

S1 Fig Funnel plot of all individual study in plasma concentrations of hsCRP. (TIF)
