# Supplementary material for: Effects of Thiazolidinedione Therapy on Inflammatory Markers of Type 2 Diabetes: A Meta-Analysis of Randomized Controlled Trials
Source: PLoS One. 2015 Apr 21;10(4):e0123703. doi: 10.1371/journal.pone.0123703 (PMC4405205; doi:10.1371/journal.pone.0123703)
Supplement: S1 PRISMA Checklist — (DOC) [file pone.0123703.s001.doc]

| **Section/topic** | **#** | **Checklist item** | **Reported on page #** |
| --- | --- | --- | --- |
| **TITLE** | | |  |
| Title | 1 | Identify the report as a meta-analysis. | 1 |
| **ABSTRACT** | | |  |
| Structured summary | 2 | **Background** Inflammation is a common feature in patients with type 2 diabetes mellitus (T_2_DM). This meta-analysis aimed to assess the influence of thiazolidinedione (TZD) therapy on the circulating levels of inflammatory markers in patients with T_2_DM.  **Methods and Results** We searched the databases Medline, Embase, ScienceDirect, Web of Science, SpringerLink, and the Cochrane Library for randomized controlled trials (RCTs) that examined the effects of thiazolidinedione vs. a placebo on patients with T_2_DM. The main outcomes were absolute changes in levels of circulating inflammatory markers. Twenty-seven RCTs were included and data were analyzed using a fixed-effect model or a random-effect model based on heterogeneity. Pooled results indicated that circulating levels of high-sensitivity C reactive protein (hsCRP; SMD = –0.65, 95% CI = –0.98 to –0.32, *p* < 0.01), monocyte chemoattractant protein-1 (MCP-1; WMD = –54.19, 95% CI = –73.86 to –34.52, *p* < 0.01), von Willebrand factor% (vWF%; WMD = –8.18, 95% CI = –13.54 to –2.81, *p* < 0.01), fibrinogen (SMD = –0.26, 95% CI = –0.41 to –0.11, *p* < 0.01) and E-selectin(WMD = –3.57, 95% CI = –5.59 to -1.54, *p<*0.01) were significantly decreased after TZD therapy. However, interleukin-6 (IL-6), matrix metalloproteinase-9 (MMP-9), soluble CD40 ligand, plasminogen activator inhibitor 1 (PAI-1) and intercellular adhesion molecule (ICAM-1) were not significantly affected. Subgroup analyses of PAI-1, vWF% and fibrinogen in terms of trial drugs showed significant reductions for rosiglitazone (all *p* valuses< 0.05), but not pioglitazone treatment .Conversely, the E-selectin (*p* < 0.01) lowering effect only existed in the pioglitazone group. Further, rosiglitazone and pioglitazone treatment reduced serum hsCRP and MCP-1 but had no marked effects on MMP-9, IL-6 and ICAM-1.  **Conclusions** Limited evidence suggested that TZD therapy had anti-inflammatory property that might contribute to its beneficial effect on inflammatory state in patients with type 2 diabetes. | 2 |
| **INTRODUCTION** | | |  |
| Rationale | 3 | Inflammatory state is a known contributor to the development of insulin resistance (IR) and vascular damage. The presence of activated circulating pro-inflammatory markers, such as high sensitivity C-reactive protein (hsCRP) and interleukin-6 (IL-6), systemic release of pro-thrombotic markers, such as plasminogen activator inhibitor 1 (PAI-1), and increased markers of endothelial dysfunction, such as E-selectin, are involved in the pathogenesis of vascular dysfunction [1–3] and IR [4,5]. Increased plasma concentrations of these inflammatory cytokines may indicate a significant increase in the risk of vascular damage and IR.Peroxisome proliferator-activated receptor (PPAR) γ agonists, also called thiazolidinediones (TZDs), along with the derivatives pioglitazone and rosiglitazone, are among the main classes of oral anti-diabetic drugs. Currently, they are extensively used worldwide [6]. TZDs have shown potential retrogression for type 2 diabetes and prolonged glycemic control by increasing insulin sensitivity in the liver, muscles, and fat [7]. Studies have also focused on the improvement of vascular dysfunction [8,9]. Results of animal and large prospective trials have indicated that rosiglitazone and pioglitazone exhibit anti-inflammatory properties [10,11,12,13]. Considering that inflammatory processes are dysregulated in the pathogenesis of IR and vascular damage, we proposed that TZD therapy could improve IR and vascular damage by suppressing plasma inflammatory cytokines. However, the effects of TZD treatment on these molecules remain inconclusive. | 3 |
| Objectives | 4 | To investigate the effects of TZD therapy on the serum levels of cytokines. | 3 |
| **METHODS** | | |  |
| Protocol and registration | 5 | NO. |  |
| Eligibility criteria | 6 | Studies were eligible for the present meta-analysis if they satisfy the following criteria: (1) human intervention studies with a prospective, randomized, and placebo-controlled trial (regardless of sample size); (2) analysis on adult patients with established type 2 diabetes and who were subjected to oral TZD (pioglitazone or rosiglitazone) therapy or placebo (we adopted the criteria established by the World Health Organization and the American Diabetes Association for the diagnosis of type 2 diabetes: fasting glucose >126 mg/dl (7.0 mmol/L) or 2 h blood glucose >200 mg/dl (11.1 mmol/L); (3) at least one of the following circulating cardiovascular risk markers was included and allowed calculation of the net change: hsCRP, matrix metalloproteinase-9 (MMP-9), monocyte chemoattractant protein (MCP)-1, IL-6, soluble CD40 ligand (sCD40L), von Willebrand factor% (vWF%), PAI-1, fibrinogen, E-selectin, and intercellular adhesion molecule (ICAM)-1; and (4) full-length articles.. | 4 |
| Information sources | 7 | We searched Medline, Embase, [ScienceDirect](http://lib.ujs.edu.cn/gczy/Statistic1.asp?ID=30), [Web of Science](http://lib.ujs.edu.cn/gczy/Statistic1.asp?ID=188), [SPRINGER LINK](http://lib.ujs.edu.cn/gczy/Statistic1.asp?ID=22), and Cochrane's library from [January](http://dict.cn/January) 2000 until [January](http://dict.cn/January) 2015 without language restrictions. | 3 |
| Search | 8 | Terms used for the search were “thiazolidinediones”, “TZDs”, “Peroxisome proliferator-activated receptor γ agonist”, “PPAR γ agonist”, “pioglitazone” and “rosiglitazone” and they were paired with the following terms “inflammation”, “cardiovascular risk marker”, and “thrombotic marker”. The search was limited to clinical trials. | 4 |
| Study selection | 9 | We searched Medline, Embase, [ScienceDirect](http://lib.ujs.edu.cn/gczy/Statistic1.asp?ID=30), [Web of Science](http://lib.ujs.edu.cn/gczy/Statistic1.asp?ID=188), [SPRINGER LINK](http://lib.ujs.edu.cn/gczy/Statistic1.asp?ID=22), and Cochrane's library from [January](http://dict.cn/January) 2000 until [January](http://dict.cn/January) 2015 without language restrictions. Terms used for the search were “thiazolidinediones”, “TZDs”, “Peroxisome proliferator-activated receptor γ agonist”, “PPAR γ agonist”, “pioglitazone” and “rosiglitazone” and they were paired with the following terms “inflammation”, “cardiovascular risk marker”, and “thrombotic marker”. The search was limited to clinical trials. The lists of original and review articles were analyzed using a manual approach. |  |
| Data collection process | 10 | Data extraction was performed by 2 authors , and the results were compiled. Disagreement was resolved by consensus and by opinion of a third author if necessary. |  |
| Data items | 11 | The following data were extracted:baseline characteristics (lead author, publication year, study design, sample size, mean age),treatment regimen (dose of pioglitazone or rosiglitazone, composition of placebo and intervention duration) . If the study provided interquartile ranges(IQRs) and medians instead of means and (standard deviations) SDs, we imputed the means and SDs as previously described. |  |
| Risk of bias in individual studies | 12 | The quality of the studies was assessed by focusing on randomization procedures, random number generation, double-blinding procedures, information on withdrawals, and allocation concealment.Studies scored 1 point for each of the areas addressed, with a potential score between 0 and 5. High-quality RCTs scored ≥3 points, whereas low-quality RCTs scored< 3 points, according to a modified Jadad score. |  |
| Summary measures | 13 | The significance of the net changes was calculated by using the weight mean difference(WMD) or standardized mean difference (SMD). |  |
| Synthesis of results | 14 | 95% confidence intervals (CI) with fixed-effect or random-effect models. The heterogeneity of intervention effects among studies was tested by using Cochrane’s test, and significant heterogeneity was considered if p<0.1. The I^2^ statistic was also examined, where I^2^ values of 25%, 50%, and 75% indicated low, moderate, and high degrees of heterogeneity, respectively. |  |

Page 1 of 2

| **Section/topic** | **#** | **Checklist item** | **Reported on page #** |
| --- | --- | --- | --- |
| Risk of bias across studies | 15 | Visual inspection of funnel plots appeared symmetric. We also used Egger’s test to check for potential publication bias. |  |
| Additional analyses | 16 | Sensitivity analysis was made by excluding studies whose data were imputed from median, IQRs and an open-labeled study. |  |
| **RESULTS** | | |  |
| Study selection | 17 | A total of 2,377 studies were initially identified; among these studies, 2,320 were excluded after titles and abstracts were screened. The full texts of the 57 remaining studies were analyzed. Among these 57 studies, 30 were excluded because of the following reasons: first, 11 studies provided insufficient data on related outputs; second, 5 studies did not include an appropriate control group; third, 3 studies used an ineligible study design; fourth, participants in 7 studies were not patients with type 2 diabetes . Finally, the endpoints were not relevant in 3 studies and 1 study reported replicated data . |  |
| Study characteristics | 18 | A total of 27 RCTs, including 5 open-labeled randomized trials (19, 25, 28, 38 and 40), were eligible for the present meta-analysis (Table 1). 11 of the observational studies (15, 16, 19, 21-24, 29, 32, 36 and 38) were used rosiglitazone as the primary source of TZDs; in the other studies (17, 18, 20, 25- 28, 30, 31, 33-35, 37, 39-41), pioglitazone was used. Pioglitazone and rosiglitazone doses varied from 15 mg/d to 45 mg/d and from 4 mg/d to 8 mg/d, respectively. Among the 27 studies, 7 investigated the effects of TZDs on type 2 diabetic patients with coronary artery disease (15, 16, 20, 27, 28, 29, and 33). 12 studies examined type 2 diabetic subjects (19, 21, 22, 24, 30-32 and 36-40) only, and the remaining studies were conducted on individuals with a combination of type 2 diabetes and one of the following conditions: dislipidemia (17); obesity (18 and 35); asymptomatic carotid stenosis (23 and 34); chronic kidney disease (24); atherosclerosis (26) and kidney transplant(41). |  |
| Risk of bias within studies | 19 | The quality scores (Table 1) of these RCTs varied from 3 to 5 (maximum score). A total of 10 studies were classified as high quality with a Jadad score of 4 or 5 (15, 16, 18, 21, 24, 33, 35, 37, 39 and 41) and 17 studies yielded a Jadad score of 3 (17, 19, 20, 22, 23, 25–32, 34, 36, 38 and 40) . |  |
| Results of individual studies | 20 | Results of individual studies could be derived from the forest plots represented in the paper. |  |
| Synthesis of results | 21 | **Effects of TZD therapy on the plasma concentrations of pro-inflammatory markers**  22 studies with 2098 patients reported the effects of TZD therapy on hsCRP levels (SMD = –0.65, 95% CI = –0.98 to –0.32, *p* < 0.01; heterogeneity test: chi-square = 244.97, *I*^2^ = 91%, *p* < 0.01; Fig. 2 A). The hsCRP-lowering effect was consistent in both subgroups: rosiglitazone [subtotal SMD = –0.90, 95% CI = –1.64 to –0.16, *p* = 0.02, *I*^2^ = 94%, 7 trials, *n =* 650] and pioglitazone [Subtotal SMD = –0.54, 95% CI = –0.92 to –0.16, *p* < 0.05, *I*^2^ = 90%, 15 trials, *n =* 1448]. Five studies were excluded for sensitivity analysis because of the following reasons: hsCRP data were imputed from median [15], IQRs [26], and three open-labeled studies [25, 38 and 40]. It also suggested a significant lowing effect (SMD = –0.36, 95% CI = –0.62 to –0.09, *p <*0.01).  In a pooled analysis of seven studies with 725 patients, the circulating levels of IL-6 were not significantly reduced (SMD = –0.45, 95% CI = –1.14 to 0.24, *p* = 0.20; heterogeneity test: chi-square = 88.91, *I*^2^ = 93 %, *p*<0.01; Fig. 2B). The IL-6 lowering effect was not observed in both subgroups: rosiglitazone [Subtotal SMD = –0.07 , 95% CI = –0.29 to 0.16, *I*^2^ = 0%, *p* = 0.55, 3 trials, *n =* 303] and pioglitazone [Subtotal SMD = -1.5, 95% CI = –3.08 to 0.07, *p* = 0.06, 4 trials, *n =* 422]. Three studies were excluded for sensitivity analysis because IL-6 data were imputed from median [15] and IQRs [24,40]. The result of the sensitivity analysis indicated that these three studies had no effect (SMD = -0.26, 95% CI = –0.75 to 0.23, *p* = 0.29).  Eight studies with 720 patients were pooled in terms of MMP-9 (WMD = –13.06, 95% CI = –27.18 to 1.05, *p =* 0.07; heterogeneity test: chi-square = 12.90, *I*^2^ = 46%, *p* = 0.07; Fig. 2C). There was no significant reduction in both subgroups: rosiglitazone [Subtotal WMD = –11.72, 95% CI = –25.24 to 1.79, *I*^2^ = 0%, *p* = 0.09, 3 trials, *n =* 293] and pioglitazone [Subtotal WMD = –32.43, 95% CI = –74.06 to 9.20, *I*^2^ = 57%, *p =*0.13, 5 trials, *n =* 427].Three studies were excluded for sensitivity analysis because of the following reasons: MMP-9 data were imputed from median [15] and two open-labeled studies [25,40]. They made no difference to the result of TZDs treatment (WMD = –9.61, 95% CI = –25.01 to 5.80, *p* = 0.22).  No significant reduction was found in sCD40 L concentration (WMD = –0.37, 95% CI = –0.80 to 0.05, *p* = 0.09; heterogeneity test: chi-square = 4.11, *I*^2^ = 27%, *p* = 0.25; Fig. 2D) based on the pooled results of four studies with 351 patients who were only in the pioglitazone group. The result of the sensitivity analysis, excluding one study in which sCD40L data were imputed from an open-labeled study [25], was not significant either (WMD = –0.50, 95% CI = –1.02 to 0.01, *p* = 0.06).  All of the four studies with 346 patients were pooled in terms of MCP-1 (WMD = –54.19, 95% CI = –73.86 to –34.52, *p* < 0.01; heterogeneity test: chi-square = 5.79, *I*^2^ = 48%, *p* = 0.12; Fig. 2E). The lowering effect was consistent in both subgroups: rosiglitazone [Subtotal WMD = –75.04, 95% CI = –102.27 to –47.81, *I*^2^ = 0%, *p* < 0.01, 2 trials, *n =* 81] and pioglitazone [Subtotal WMD = –31.43, 95% CI = –59.87 to –2.98, *I*^2^ = 0%, *p* = 0.03, 2 trials, *n =* 265]. The sensitivity analysis, excluding one study in which MCP-1 data were imputed from an open-labeled study [25], did not affect the result (WMD = –68.01, 95% CI = –92.17 to –43.85, *p* < 0.01).  **Effects of TZD therapy on the plasma concentrations of pro-thrombotic markers**  Six studies with 589 patients reported the effects of TZD therapy on vWF% levels (WMD = –8.18, 95% CI = –13.54 to –2.81, *p* < 0.01; heterogeneity test: chi-square = 1.91, *I*^2^ = 0.0%, *p* = 0.86; Fig. 3A). However, differences were observed between the subgroups rosiglitazone [Subtotal WMD= –7.95, 95% CI = –14.15 to –1.75, *I*^2^ = 0%, *p* = 0.01, 4 trials, *n =* 370] and pioglitazone [Subtotal WMD = –8.85, 95% CI = –19.56 to 1.85, *I*^2^ = 0%, *p* = 0.11, 2 trials, *n =* 219].The sensitivity analysis, excluding two studies in which vWF% data were imputed from an open-labeled study [25] and IQRs [21], did not affect the result (WMD = –8.29, 95% CI = –14.62 to –1.96, *p* = 0.01).  No significant reduction was found in the PAI-1 concentrations (WMD = –1.52, 95% CI = –3.31 to 0.26, *p* = 0.09; heterogeneity test: chi-square = 6.29, *I*^2^ = 21%, *p* = 0.28; Fig. 3B) based on the pooled results from six studies with 542 patients. However, different effects were observed between the subgroups rosiglitazone [Subtotal WMD = –5.88, 95% CI = –10.14 to –1.62, *I*^2^ = 0%, *p* < 0.01, 2 trials, *n =* 233] and pioglitazone [Subtotal WMD = –0.60, 95% CI = –2.56 to 1.37, *I*^2^ = 0%, *p* = 0.55, 4 trials, *n =* 309]The sensitivity analysis, excluding two studies in which PAI-1 data were imputed from an open-labeled study [25] and median [15], suggested that the effect of TZDs therapy was not significant either (WMD = –0.88, 95% CI = –4.69 to 2.92, *p* = 0.65).  Four studies with 664 patients were pooled in terms of fibrinogen (SMD = –0.26, 95% CI = –0.41 to –0.11, *p* < 0.01; heterogeneity test: chi-square = 4.01, *I*^2^ = 25%, *p* = 0.26; Fig. 3 C). However, when it comes to the subgroup analysis, different results were observed: rosiglitazone [Subtotal SMD = –0.35, 95% CI = –0.53 to –0.17, *I*^2^ = 0%, *p* < 0.01, 2 trials, *n =* 491] and pioglitazone [Subtotal SMD = 0 , 95% CI = –0.30 to 0.30, 1 trial, *n =* 173].  **Effects of TZD therapy on plasma concentrations of adhesion molecules**  All of the five studies with 629 patients reported the effects of TZD therapy on E-selectin levels (WMD = –3.57, 95% CI = –5.59 to -1.54, *p<*0.01; heterogeneity test: chi-square = 5.22, *I*^2^ = 23%, *p* = 0.27; Fig. 4A). The effects were not consistent when the subgroup analysis based on different trial drugs were considered: rosiglitazone [Subtotal WMD = –2.42, 95% CI = –14.08 to 9.25, *I*^2^ = 78%, *p* = 0.68, 2 trials, *n =* 233] and pioglitazone [Subtotal WMD = –3.91, 95% CI = –5.01 to -2.81, *I*^2^ = 0%, *p*<0.01, 3 trials, *n =* 396].  No significant reduction was determined in the ICAM-1 concentration (SMD = 0.30, 95% CI = –0.07 to 0.66, *p* = 0.11; Fig. 4B). The effects of the subgroup analysis were consistent: rosiglitazone [Subtotal SMD = 0.43, 95% CI = –0.57 to 1.42, *I^2^* = 87%, *p* = 0.40, 2 trials, *n* = 233] and pioglitazone [Subtotal SMD = 0.26, 95% CI = –0.20 to 0.72, *I*^2^ = 81%, *p* = 0.26, 3 trials, *n =* 493]. The sensitivity analysis, excluding one study in which ICAM-1 data were imputed from an open-labeled study [25], revealed a not significant effect either (SMD = 0.40, 95% CI = –0.03 to 0.82, *p* = 0.07). |  |
| Risk of bias across studies | 22 | Visual inspection of funnel plots appeared symmetric. We also used Egger’s test to check for potential publication bias, which showed no evidence of publication for the outcomes of hsCRP (P=0.152). |  |
| Additional analysis | 23 | The results of sensitivity analysis were represented in Item 21. |  |
| **DISCUSSION** | | |  |
| Summary of evidence | 24 | The present meta-analysis included 27 trials which investigated the effects of TZD therapy on the plasma concentrations of cytokines in patients with type 2 diabetes. Significant reductions in the majority of these cytokines, such as hsCRP, MCP-1, vWF%, fibrinogen and E-selectin were observed in the TZD group compared with those of the placebo group among patients with type 2 diabetes. The levels of IL-6, MMP-9, sCD40L , PAI-1 and ICAM-1 showed no significant change. In the subgroup analyses based on different trial drugs, the lowering effects of PAI-1, vWF%, and fibrinogen were only observed in the rosiglitazone group. E-selectin level was significantly decreased only in the pioglitazone group. The serum hsCRP and MCP-1 revealed marked reductions in each group. There was no remarkable change in plasma IL-6, MMP-9 and ICAM-1 in both groups. |  |
| Limitations | 25 | The potential limitations of our meta-analysis are as follows: first ,many of these trials were small and short-term, correspondingly the confidence intervals for some cardiovascular risk markers were wide, resulting inconsiderable uncertainty about the magnitude of the observed reduction; second, the subjects’ health status, lifestyle and the basical oral antidiabetic treatment were different which might be important source of heterogeneity; third, we did not have to access to original source data of the individual participants. Besides, a meta-analysis is always regarded as deficient in authority than a large prospective trial designed to evaluate the outcome of interest. |  |
| Conclusions | 26 | Our meta-analysis that pooled the limited number of RCTs indicated that rosiglitazone and pioglitazone therapy administered to patients with type 2 diabetes could reduce the circulating concentrations of several specific inflammatory markers. These results suggested that TZD treatment of patients with type 2 diabetes could elicit anti-inflammatory effects. |  |
| **FUNDING** | | |  |
| Funding | 27 | The authors thank the field workers for their contribution and the participants for their cooperation. This project was supported by the Natural Science Foundation and the development health engineering of Jiangsu Province (BK2011486, LJ201116) and National Natural Science Foundation of China (81170279, 81370409) and Key Laboratory of Cardiovascular disease of Zhenjiang (SS2012002). The funders had no role in study design, data collection and analysis, decision to publish, or preparation of the manuscript. |  |

*From:*  Moher D, Liberati A, Tetzlaff J, Altman DG, The PRISMA Group (2009). Preferred Reporting Items for Systematic Reviews and Meta-Analyses: The PRISMA Statement. PLoS Med 6(6): e1000097. doi:10.1371/journal.pmed1000097

For more information, visit: **www.prisma-statement.org**.

Page 2 of 2
