# Supplementary figures and images for: Effects of Thiazolidinedione Therapy on Inflammatory Markers of Type 2 Diabetes: A Meta-Analysis of Randomized Controlled Trials
Source: PLoS One. 2015 Apr 21;10(4):e0123703. doi: 10.1371/journal.pone.0123703 (PMC4405205; doi:10.1371/journal.pone.0123703)

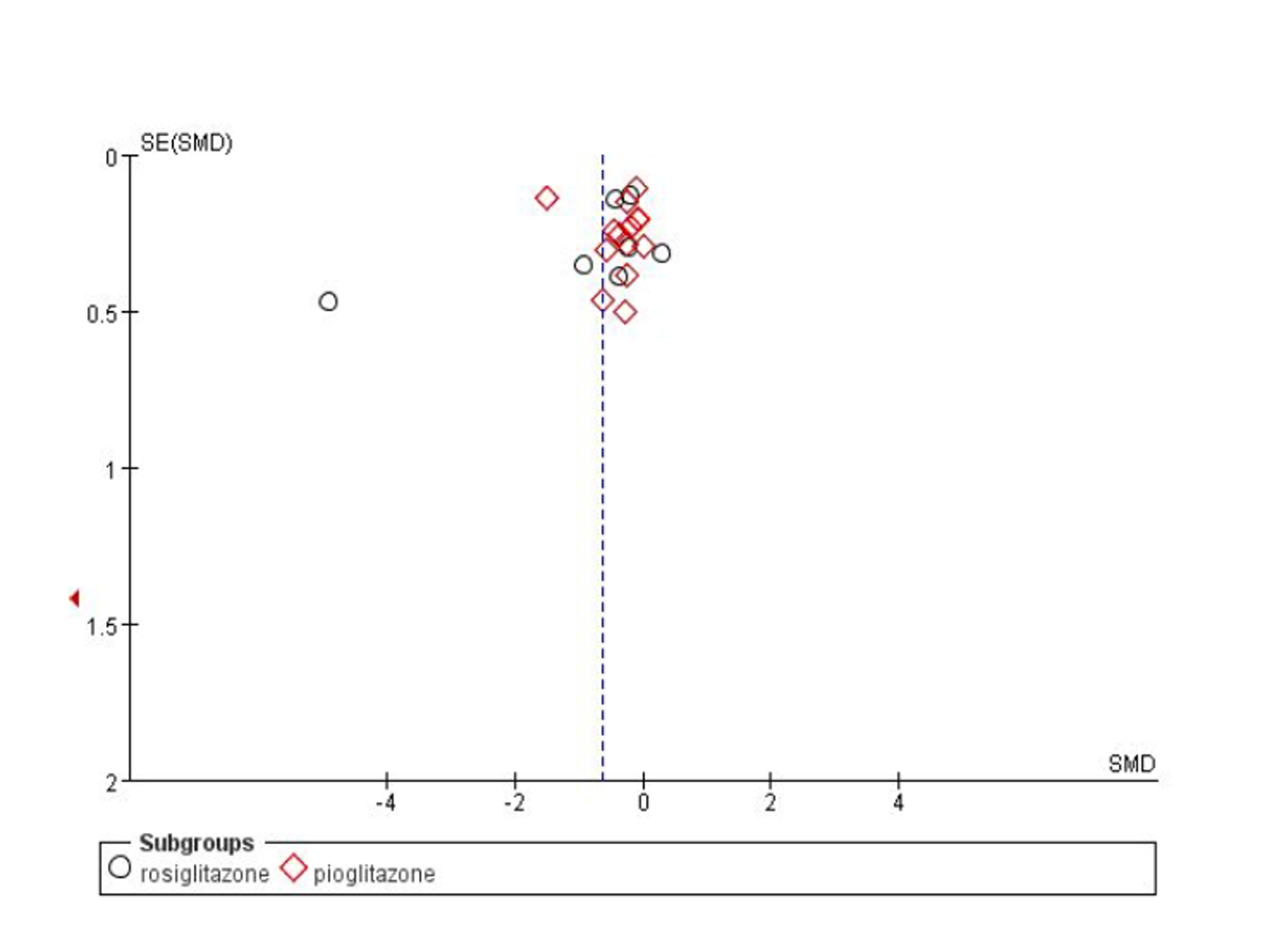

Supplement: S1 Fig — (TIF) [file pone.0123703.s002.tif]
